# Performance of newly developed body mass index cut-off for diagnosing obesity among Ethiopian adults

**DOI:** 10.1186/s40101-019-0205-2

**Published:** 2019-10-26

**Authors:** Makeda Sinaga, Tilahun Yemane, Elsah Tegene, David Lidstrom, Tefera Belachew

**Affiliations:** 1 Department of Nutrition and Dietetics, Faculty of Public Health, Jimma University, Po. Box 378, Jimma, Ethiopia; 2 Faculty of Health Sciences, Department of Laboratory Sciences, Jimma University, Jimma, Ethiopia; 3 Department of Internal Medicine, Faculty of Medicine, Jimma University, Jimma, Ethiopia; 4 Population Studies Centre, Brown University, Providence, USA

**Keywords:** Validity, Body mass index, Cut-off, Obesity, Ethiopia

## Abstract

**Background:**

Obesity is defined as unhealthy excess body fat, which increases the risk of premature mortality from noncommunicable diseases. Early screening and prevention of obesity is critical for averting associated morbidity, disability, and mortality. Ethiopia has been using the international (WHO’s) BMI cut-off for diagnosing obesity even though its validity among Ethiopian population was questioned. To address this problem, a new body mass index cut-off was developed for Ethiopian adults using population-specific data. However, its performance in diagnosing obesity has not been validated. Therefore, this study determined the performance of the newly developed Ethiopian and World Health Organization (WHO) BMI cut-offs in detecting obesity among Ethiopian adults.

**Methods:**

A cross-sectional study was carried out among 704 employees of Jimma University from February to April 2015. The study participants were selected using simple random sampling technique based on their payroll. Data on sociodemographic variables were collected using an interviewer-administered structured questionnaire. Anthropometric parameters including body weight and height were measured according to WHO recommendation. Body fat percentage (BF%) was measured using the air displacement plethysmography (ADP) after calibration of the machine. The diagnostic accuracy of the WHO BMI cut-off (≥ 30 kg/m^2^) for obesity in both sexes and Ethiopian BMI cut-off (> 22.2 kg/m^2^ for males and >  24.5 kg/m^2^ for females) were compared to obesity diagnosed using ADP measured body fat percentage (> 35% for females and >  25% for males). Sensitivity, specificity, predictive values, and kappa agreements were determined to validate the performance of the BMI cut-offs.

**Results:**

In males, WHO BMI cut-off has a sensitivity of 5.3% and specificity of 99.4% (Kappa = 0.047) indicating a slight agreement. However, the Ethiopian cut-off showed a sensitivity of 87.5% and specificity of 87.7% (Kappa = 0.752) indicating a substantial agreement.

Similarly, in females, the WHO BMI cut-off showed a sensitivity of 46.9%, while its specificity was 100% (Kappa = 0.219) showing a fair agreement. The Ethiopian BMI cut-off demonstrated a sensitivity 80.0% and a specificity 95.6% (Kappa = 0.701) showing a substantial agreement. The WHO BMI cut-off underestimated the prevalence of obesity by a maximum of 73.7% and by a minimum of 28.3% among males, while the values for underestimation ranged from 31.4–54.1% in females. The misclassification was minimal using the newly developed Ethiopian BMI cut-off. The prevalence of obesity was underestimated by a maximum of 9.2% and overestimated by a maximum of 6.2%. The WHO BMI cut-off failed to identify nearly half (46.6%) of Ethiopian adults who met the criteria for obesity using BF% in the overall sample.

**Conclusions:**

The findings suggest that WHO BMI cut-off (≥ 30 kg/m^2^) is not appropriate for screening obesity among Ethiopian adults. The newly developed Ethiopian BMI cut-off showed a better performance with excellent sensitivity, specificity, predictive values, and agreement indicating the diagnostic significance of it use as a simple, cost-effective, and valid indicator in clinical and community setups.

## Introduction

Obesity is associated with increased risk numerous co-morbid conditions such as type 2 diabetes melitus, systemic hypertension, dyslipidemia, obstructive sleep apnea, osteoarthritis, depression, gout, nonalcoholic liver disease, reproductive-endocrine disorders and several cancers [1–5]. The prevalence of obesity is rapidly increasing in the world exacting a heavy loss both socially and economically [6–8].

As a result of an epidemiological transition related to increased urbanization, westernization, and globalization, many sub-Saharan African countries are experiencing lifestyle and behavioral changes such as unhealthy diet, physical inactivity, and increased tobacco and alcohol use leading to an increasing prevalence of obesity [9–14]. Consequently, evidence suggests that there has been an increasing prevalence of metabolic syndrome (MetS) among populations in sub-Saharan African countries including Ethiopia over the past decades [15–18]. Body weight guidelines are useful for practitioners to screen patients for excessive adiposity and prescribe treatment for patients with overweight [19]. World Health Organization (WHO) recommends body mass index (BMI) ≥ 30 kg/m^2^ to be used as a simple indicator of obesity in adults [20] in all countries that do not have locally appropriate cut-off.

However, the definition of obesity based on this cut-off has been challenged due to variations in the relationships between the body fat percentage and BMI in different populations [21–23]. Accurate determination of obesity has become exceedingly important because of major health threats posed by excess adiposity, which could lead to misleading conclusions about obesity and associated health status [21–23].

A meta-analysis of different studies revealed that the above BMI cut-off failed to identify half of the people with excess body fat percentage [24, 25]. This has an effect on the validity of BMI for screening the risk of type 2 diabetes and cardiovascular disease [26]. Another meta-analysis of studies on the relationship between body fat percentage measured and estimated based on BMI from Caucasian equation in different ethnic groups showed that BMI underestimates body fat percentage among Ethiopians [27]. Studies based on data generated from few Ethiopians with limited ethnic representation indicated that the international (WHO’s) BMI  cut-off is inappropriate for Ethiopians [27–29].

Although there are several advanced obesity (body fat percentage) measurement techniques including air displacement plethysmography (ADP), bioelectrical impedance analyses (BIA), dual-energy X-ray absorptiometry (DEXA), hydrostatic weighing, and other multicompartment models, they are too expensive and not available for routine service level use in developing countries including Ethiopia. To overcome this problem, a new BMI cut-off was developed for Ethiopians based locally appropriated data using large ethnically representative sample [30]. However, the performance of this new cut-off in diagnosing obesity was not assessed. The use of the cut-off by service providers, researchers, and policy makers requires an evidence on its validity. In this study, we compared obesity measured using the newly developed Ethiopian BMI cut-off [30] and WHO's BMI cut-off [20, 31, 32] with obesity determined based on the body fat percentage measured using ADP (gold standard) [33].

## Methods and materials

### Study setting and participants

The study was conducted from February to March 2015 in Jimma University, which is a public institution situated 357 km southwest of Addis Ababa. The university has two institutes and six colleges housing a total of 1341 academic and 5444 administrative staff. All administrative and academic staff of Jimma University who were actively working and not away for more than one week during the recruitment period were included in the study. Those who had a physical disability including deformity (kyphosis or scoliosis), limb deformity preventing them from standing erect, pregnant women, and those who were seriously ill were excluded from the study.

An institution-based cross-sectional study was conducted among 704 employes of Jimma University randomly selected using the payroll as a sampling frame. The sample size was determined using sensitivity estimation formula as presented elsewhere [30]. The staff of Jimma University was stratified by sex, and the study participants were selected from each stratum randomly using proportional to size (PPS) allocation method.

### Measurements

Data were collected by five trained clinical nurses who were recruited based on their qualification and prior experience in data collection. A 5-day training was given to data collectors before the actual survey. Supervisors made a close follow-up of measurements and interviews during the field work.

Height and weight were measured according to the WHO STEPS procedure [34]. A stadiometer (Seca Germany) was used to measure the height of the study participants to the nearest 0.1 cm with the subjects positioned at the Frankfurt Plane and the four points (heel, calf, buttocks, and shoulder) touching the vertical stand of the stadiometer and their shoes taken off. Before starting the measurements, the stadiometer was checked using a calibration rod. An electric-powered digital scale (Seca Germany) was used to measure the weight to the nearest 0.1 kg with the subjects wearing light clothes and shoes taken off. An object of a known weight was used to check the validity of the scale every morning. All anthropometric measurements were done in triplicate, and the average value was used for further analyses.

Body fat percentage was measured using air displacement plethysmography (ADP) following recommended procedures [25, 35–38]. The air displacement plethysmography was calibrated for weight and volume using an adult cylinder of known volume and weight. The procedure for measurement of the body fat percentage was thoroughly explained to the study participants. Subjects wore a similar pant and took off all other clothes, and those with long hair wore swimmer’s cape. Strict instruction was given to the study participants to come without eating or drinking within 2 h for body fat percentage measurement. Body fat measures were obtained as a printout or digitally within 2 min. Quality-control measures were also performed before anthropometric measurements. Standardization of procedures, training of data collectors, and validation and calibration of equipment were performed before beginning the data collection.

To validate the BMI cut-off, the obesity (adiposity) determined using Ethiopian BMI cut-off > 22.2 kg/m^2^ (for males) and > 24.5 kg/m^2^ (for females) [30] and WHO BMI ≥ 30 kg/m^2^ cut-off [39] were compared with obesity defined by body fat percentage measured using air displacement plethysmography (ADP) [35–38]. Accordingly, obesity was defined as body fat percentage (BF %) > 25% for males and > 35% for females [20, 31, 40, 41]. Kappa was calculated as a measure of agreement for binary variables. Measures of validity including sensitivity, specificity, positive, and negative predictive values were computed as measures of validity. Body mass index was computed as the ratio of weight in kilograms to height in meter squared.

### Definition of terms

In this study, obesity was defined using different measures as follows:

- BM ≥30 kg/m^2^ according to WHO cut-off) [20, 31, 32].
- BMI > 22.2 kg/m^2^ for males and > 24.5 kg/m^2^ for females according to the new Ethiopian cut-off [30].
- ADP measured body fat percentage > 25% in males and > 35% in females [33].

### Data processing and analysis

Data were entered into Epidata version 3.1 and exported to SPSS for windows version 20.0 for cleaning and analyses. The data were checked for normality of continuous variables using QQ-plot. Descriptive analysis was used to describe the study subjects and presented using tables and figures. Validity measures including sensitivity, specificity, and positive and negative predictive values were determined for Ethiopian cut-off and for the WHO cut-off. In addition, agreement between the Ethiopian cut-off and the WHO cut-off with the gold standard (obesity measured using ADP-generated body fat percentage) were determined using Kappa statistics.

## Results

### Background and characteristics

Out of the 704 study participants, more than half (56.4%) were females, and larger proportion (38.2%) was in the age group between 20 and 30 and followed by those in the age group of 31–40 years. The mean age (± sd) was 34.7 (± 9.5) and 36.5 (± 9.2) years for males and females, respectively.

Regarding ethnicity, larger proportion of the study participants was Oromo (36.2%) followed by Amhara (30.3). The mean (± sd) weight was 67.0 (± 11.7) and 62.3 (± 12.9) kg for males and females, respectively, while the average (± SD) height was 171.8 (± 13.4) and 157.1 (± 8.5) cm for males and females, respectively. The mean BMI was higher for females (25.3 kg/m^2^) compared to males (22.5 kg/m^2^). Likewise, the measured body fat percentage (mean ± SD) was higher for females (38.5%) compared to males (23.9%) (Table 1).

**Table 1:** Background and anthropometric characteristics of the study participants (*n* = 704)

Characteristics	*n*	Percent
Sex		
Female	397	56.4
Male	307	43.6
Ethnic groups		
Oromo	255	36.2
Amhara	213	30.3
Gurage	38	5.4
Kefa	50	7.1
Others (Sidama,Wolaita, Tigre)	48	6.8
Dawero	57	8.1
Yem	43	6.1
Age group (years)		
20–30	269	38.2
31–40	250	35.5
≥ 41	185	26.3
		Mean (SD
Height (cm)		
Male	307	171.8 (13.4)
Female	397	157.1 (8.5)
Weight (kg)		
Male	307	67.0 (11.7)
Female	397	62.3 (12.9)
BMI (kg/m^2^)		
Female	397	25.3 (5.1)
Male	307	22.5 (3.9)
Total	704	24.1 (4.8)
Measured body fat mass fat % (mean ± SD)		
Female	397	38.5 (10.1)
Male	307	23.9 (9.2)

*SD* standard deviation

#### Performance of newly developed Ethiopian and WHO BMI cut-offs in diagnosing obesity

For male Ethiopian adults, obesity determined based on WHO BMI cut-off (≥ 30 kg/m^2^) highly underestimated obesity (maximum Kappa = 0.081 for age groups and Kappa = 0.064 for ethnicity). The results indicated that the BMI underestimated the prevalence of obesity by a maximum of 73.7% among males in the age group greater than 40 years and by a minimum of 28.3% among males in the age group of 20–30 years. However, the difference was minimal using the newly developed Ethiopian BMI cut-off. The prevalence of obesity was underestimated by a maximum of 9.2% in the age group greater than 40 years and overestimated by a maximum of 6.2% among those in the age group of 20–30 years (Table 2).

**Table 2:** Agreement of obesity prevalence measured by Ethiopian and WHO BMI cut-offs with obesity based on  ADP measured body fat percentage among Ethiopian adult males

Variables	*n*	Prevalence of obesity						
		(*a*) Obesity based on ADP measured body fat > 25% for males (%)	(*b*) Obesity based on BMI ≥ 30 kg/m^2^ (%)	Difference (*a*–*b*) (%)	Kappa	(*c*) Obesity based on Ethiopian BMI cut-off males > 22.2 kg/m^2^ (%)	Difference (*a*–*c*) (%)	Kappa
Age group								
20–30	145	29.7	1.4	28.30	0.019	35.9	− 6.20	0.642
31–40	86	59.3	5.8	53.50	0.081	57.0	2.30	0.809
≥ 41	76	76.3	2.6	73.70	0.017	67.1	9.20	0.775
Total	307	49.5	2.9	46.60	0.047	49.5	0.00	0.752
Ethnicity								
Oromo	149	38.3	2.0	36.30	0.064	43.0	− 4.70	0.792
Amhara	73	64.4	5.5	58.90	0.019	63.0	1.40	0.674
Gurage	16	81.2	6.2	75.00	0.030	56.2	25.00	0.458
Kefa	12	58.3	0.0	58.30	0.000	58.3	0.00	1.000
Others	28	57.1	3.6	53.50	0.054	57.1	0.00	0.708
Dawro	17	52.9	0.0	52.90	0.000	35.3	17.60	0.653
Yem	12	25.0	0.0	25.00	0.000	33.3	− 8.30	0.800
Total	307	49.5	2.9	46.60	0.047	49.5	0.0	0.752

Kappa agreement (0 = no/poor), (0.01–0.20 = slight), (0.21–0.40 = fair), (0.41–0.60 = moderate), (0.61–0.80 = substantial), and (0.81–1.00 = almost perfect) (William et al., 2011). BMI cut-off for obesity ≥ 30 kg/m^2^ is the WHO cut-off [20]

*ADP* air displacement plethysmography

In female Ethiopian adults, WHO BMI cut-off ( ≥ 30 kg/m^2^) significantly (*P* < 0.0001) underestimated obesity in all age groups and ethnicities, with little variation between the different groups. Analyses of obesity prevalence by age category showed that BMI cut-off underestimates obesity by a maximum of 54.1% among age group > 40 years and by a minimum of 31.4% among those in the age group 20–30 years.

However, the difference in the prevalence of obesity determined based on the body fat percentage and the new Ethiopian cut-off was small. The Ethiopian BMI cut-off underestimated obesity prevalence by a maximum of 12.0% among those in the age group 20–30 years and by a minimum of (10.3%) in the age groups 31–40 years (Table 3).

**Table 3:** Agreement of obesity prevalence measured by Ethiopian and WHO BMI cut-offs with obesity based on ADP measured body fat percentage among Ethiopian adult females

Variables	Prevalence of obesity							
	*n*	(*a*) Obesity based on ADP measured body fat > 35% for females (%)	(*b*) Obesity based on BMI ≥ 30 kg/m^2^ (%)	Difference in obesity (*a*–*b*) (%)	Kappa	(*c*) Obesity based on Ethiopian BMI cut-off for females > 24.5 kg/m^2^ (%)	Difference in obesity (*a*–*c*) (%)	Kappa
Age group								
20–30	124	39.5	8.1	31.40	0.237	27.4	12.10	0.697
31–40	164	76.8	23.8	53.00	0.172	66.5	10.30	0.630
≥ 41	109	78.0	23.9	54.10	0.163	65.1	12.90	0.647
Total	397	65.5	18.9	46.60	0.219	53.9	11.60	0.701
Ethnicity							9.50	
Oromo	106	64.2	20.8	43.40	0.255	54.7	12.20	0.729
Amhara	140	77.9	27.9	50.00	0.198	65.7	13.70	0.636
Gurage	22	77.3	9.1	68.20	0.057	63.6	15.80	0.680
Kefa	38	60.5	10.5	50.00	0.143	44.7	15.00	0.691
Others	20	45.0	15.0	30.00	0.355	30.0	7.50	0.688
Dawro	40	52.5	5.0	47.50	0.091	45.0	12.90	0.652
Yem	31	41.9	9.7	32.20	0.258	29.0	11.6	0.723
Total	397	65.5	18.9	46.60	0.219	53.9		0.701

Kappa agreement (0 = no/poor), (0.01–0.20 = slight), (0.21–0.40 = fair), (0.41–0.60 = moderate), (0.61–0.80 = substantial), and (0.81–1.00 = almost perfect) (William et al., 2011). BMI cut-off for obesity ≥ 30 kg/m^2^ is the WHO cut-off [20]

*ADP* air displacement plethysmography

**Fig. 1:**
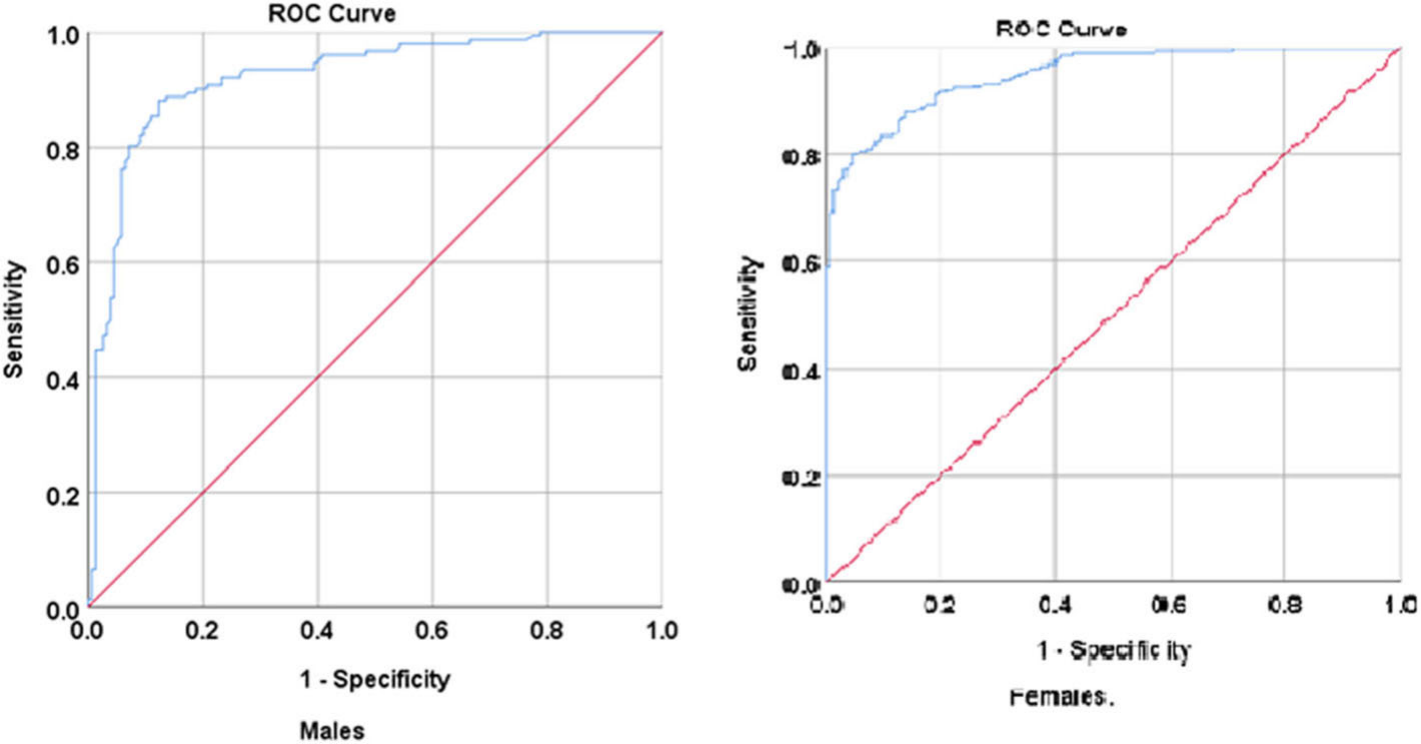
Receiver operating characteristic curve showing the diagnostic performance of BMI cut-off as compared to the body fat percentage measured by the air displacement plethysmography

Among males, it was also observed that the WHO cut-off (BMI ≥ 30 kg/m^2^) has a sensitivity of 5.3% and specificity of 99.4% (Kappa = 0.047) indicating a slight agreement. However, the Ethiopian cut-off (BMI > 22.2 kg/m^2^) showed a sensitivity of 87.5% and a specificity of 87.7% (Kappa = 0.752) indicating substantial agreement. Similarly, among females, the WHO cut-off (BMI ≥ 30 kg/m^2^). showed a sensitivity of 46.9%, while its specificity was 100%, (Kappa = 0.219) showing a fair agreement. However, the Ethiopian cut-off (BMI > 24.5 kg/m^2^) showed a sensitivity of 80.0% and a specificity of 95.6% (Kappa = 0.701) showing a substantial agreement (Table 4 and Fig. 1).

**Table 4:** Validity of BMI cut-off for detecting obesity among Ethiopian adults as compared to obesity based on the body fat percentage determined by the air displacement plethysmography (ADP)

Sex	Cut-off values	TP (*a*)	FP (*b*)	FN (*c*)	TN (*d*)	Total	Sensitivity (%)	Specificity (%)	PPV (%)	NPV (%)	Kappa	Agreement	*P*
Males	BMI ≥ 30 kg/m^2^	8	1	144	154	307	5.3	99.4	88.9	51.7	0.047	Slight	0.0160
	BMI > 22.2 kg/m^2a^	133	19	19	136	307	87.5	87.7	87.5	87.7	0.752	Substantial	< 0.0001
Females	BMI ≥ 30 kg/m^2^	75	0	185	137	397	46.9	100.0	100.0	42.5	0.219	Fair	< 0.0001
	BMI > 24.5 kg/m^2a^	208	6	52	131	397	80.0	95.6	97.2	71.6	0.701	Substantial	< 0.0001

Sensitivity = *a*/*a* + *c*, specificity = *d*/*b* + *d*, positive predictive value (PPV) = *a*/*a* + *b*, negative predictive value (NPV) = *d*/*c* + *d*. Kappa agreement (0 = no/poor), (0.01–0.20 = slight), (0.21–0.40 = fair), (0.41–0.60 = moderate), (0.61–0.80 = substantial), and (0.81–1.00 = almost perfect) (William et al., 2011). ADP measured body fat percentage > 25 for males and > 35 for females was used as a gold standard [20]. BMI cut-off for obesity ≥ 30 kg/m^2^ is the WHO cut-off [20]

*ADP* air displacement plethysmography

^a^Ethiopian sex-specific cut-off for BMI for defining obesity based on the local data

**Fig. 2:**
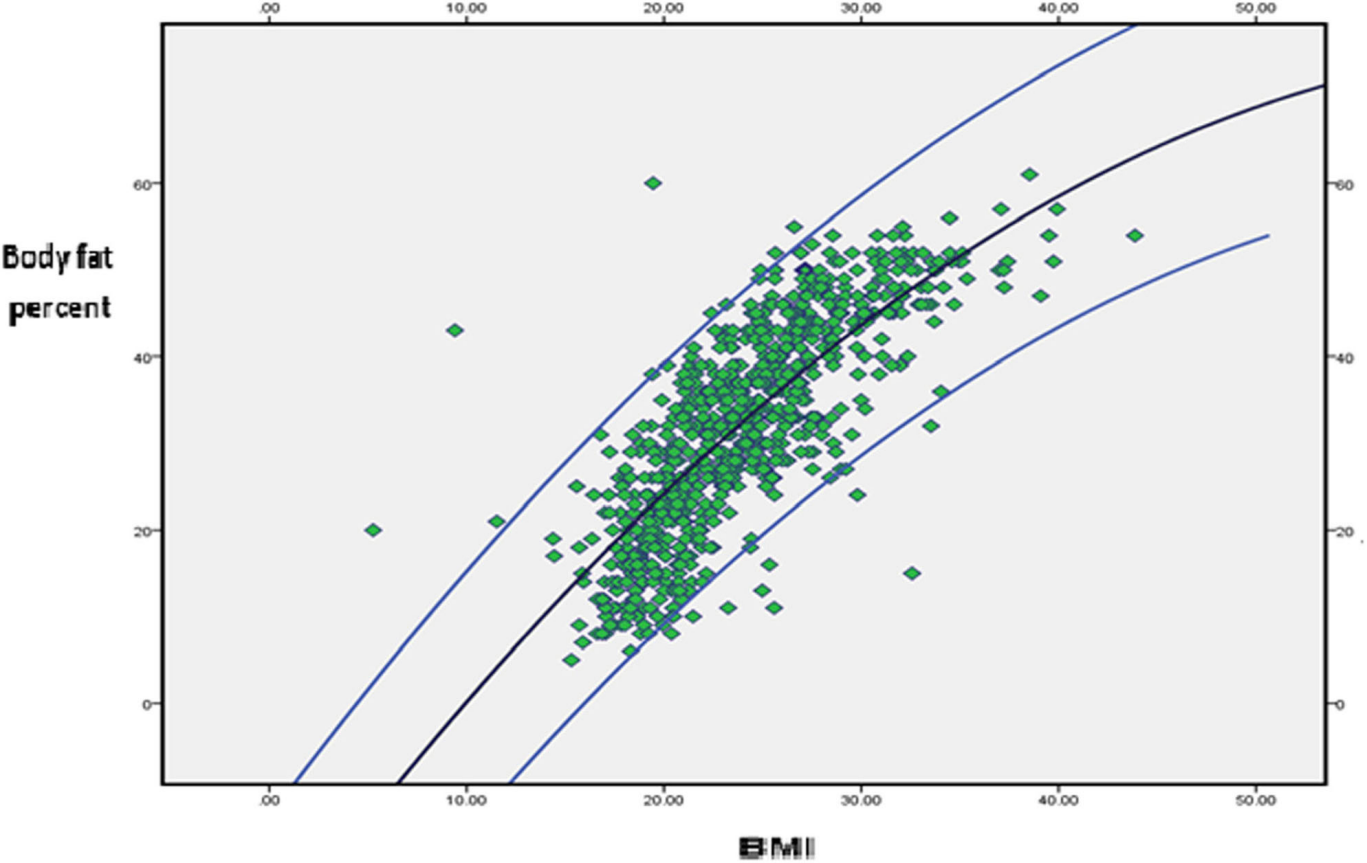
The relation between the BMI and body fat percentage among Jimma University employs

As depicted in Fig. 2, the relationship between the body fat percentage measured by ADP and BMI of Ethiopian adults is not linear. The higher the body fat percentage did not linearly translate into higher BMI values, especially for people with high body fat percentage.

## Discussion

The findings showed that the WHO BMI cut-off significantly misclassifies obesity among Ethiopian adults, regardless of age, sex, and ethnicity resulting in a significant underestimation. There was a poor to fair agreement [42] between the obesity measured using the body fat percentage and that determined using WHO BMI cut-off [32]. A similar underestimation of obesity by the international cut-off was reported among Asians [43], Syrians [44], Turkish population [45], and Chinese [46].

The fact that the relationship between the body fat percentage and BMI varies among different ethnic groups and the need for developing population-specific BMI cut-offs for obesity was recommended [27]. This disparity in detecting obesity between the BMI and measured body fat percentage could be attributed to the differences in body frames between the Ethiopians and the Caucasians on which the WHO BMI cut-off is based [32]. Ethiopians have slender body frame and more visceral fat for the same body mass index as compared to Caucasian and other Blacks [27]. Ethiopians have a higher body fat percentage compared to Caucasians [27] similar to Indians [47]. This makes the use of WHO BMI cut-off for the screening purposes unreliable and invalid for Ethiopian adults. According to the consensus of the International Diabetic Federation (IDF), developing countries should use European cut-off until they develop their cut-off based on locally relevant data [48, 49].

Conversely, there was a substantial agreement [42] between the obesity estimated based on the newly developed Ethiopian BMI cut-off [30] and the one determined based on the body fat percentage measured using ADP for all age and Ethnic groups of both sexes (Kappa = 0.75 for males and 0.70 for females). This agreement in all ethnic and age groups of both sexes indicates validity of the new cut-off in detecting obesity among Ethiopian adults. Similarly, many Asian countries [47, 50–52] had developed their own BMI cut-off for obesity based on population-specific data.

It was also observed that body fat percentage determined by ADP and BMI do not have linear relationship, among people with high body fat percentage (obese). Our data showed that as body fat increased, the relationship tends to be nonlinear. Their relationship is better described by a curvilinear pattern, which could explain the reason why there is a significant misclassification of obesity determined based on the body fat percentage and BMI for obese people in our sample. Such a nonlinear relationship was also documented by other studies [21, 53].

Despite this observation, the newly developed Ethiopian BMI cut-off showed a lower misclassification of obesity and better validity compared to the WHO cut-off. The findings imply that Ethiopian BMI cut-off could be used as simple cost-effective tool for screening and early identification of obesity with the view to promoting preventive public health actions at the population level. This study indicated that the new cut-off [30] performed very well in terms of its agreement, sensitivity, and specificity compared to the most valid measures of obesity body fat percentage.

The findings of this study have a wider practical implication in the prevention of chronic noncommunicable diseases that are currently causing a large burden of morbidity and mortality in Ethiopia [54]. Especially, as Ethiopia is growing economically and there is a rapid increase in urbanization, out of home eating, and calorie consumption [55], having such a simple, cheap, and valid tool for screening obesity enhances the efforts for prevention of obesity and related noncommunicable diseases. The results call for developing guidelines for screening and surveillance of obesity both at the community and health facility level based on the newly developed Ethiopian cut-off.

The fact that the study used body fat measured by ADP, which is reported to be very accurate [35, 37] can be considered as strength. In addition, the study indicated the fact that the international cut-off is not appropriate for Ethiopian adults, while the locally developed Ethiopian cut-off is an appropriate alternative to be used in Ethiopia. This finding is a very critical input for researchers and service providers and educators as this study is the first of its kind.

In this study, however, we acknowledge the following limitations. Although different major ethnicities were represented in the sample, it was not possible to get an adequate sample for some ethnicities. To overcome this, samples were drawn from the university, which gave an opportunity to include most of the ethnic groups. It is felt that, given these limitations, the validation done in this study could reflect the performance of both the WHO and locally generated cut-offs for Ethiopia.

## Conclusion

The study demonstrated that the Ethiopian cut-off has higher sensitivity, specificity, and predictive values and could be used as a simple cost-effective valid tool to detect obesity among Ethiopian adults. Conversely, the WHO BMI cut-off is not appropriate for screening obesity among Ethiopian adults. The findings suggest the need for using the new Ethiopian MBI cut-off for the screening of obesity among Ethiopian adults in galvanizing public health interventions to prevent obesity and associated morbidity and mortality in Ethiopia.

## Data Availability

The data used and/or analyzed during the current study are available from the corresponding author on a reasonable request.

## Abbreviations

ADP: Air displacement plethysmography
BF%: Body fat percentage
BMI: Body mass index
